# Tetramethylpyrazine: A Review of Its Antitumor Potential and Mechanisms

**DOI:** 10.3389/fphar.2021.764331

**Published:** 2021-12-16

**Authors:** Shaojie Yang, Shuodong Wu, Wanlin Dai, Liwei Pang, Yaofeng Xie, Tengqi Ren, Xiaolin Zhang, Shiyuan Bi, Yuting Zheng, Jingnan Wang, Yang Sun, Zhuyuan Zheng, Jing Kong

**Affiliations:** ^1^ Biliary Surgery (2nd General) Unit, Department of General Surgery, Shengjing Hospital of China Medical University, Shenyang, China; ^2^ Innovation Institute of China Medical University, Shenyang, China; ^3^ Department of Cardiology, Shengjing Hospital of China Medical University, Shenyang, China

**Keywords:** tetramethylpyrazine, ligustrazine, antitumor, apoptosis, metastasis, angiogenesis, chemotherapy, multidrug resistant

## Abstract

Cancer remains a major public health threat. The mitigation of the associated morbidity and mortality remains a major research focus. *From a molecular biological perspective*, cancer is defined as uncontrolled cell division and abnormal cell growth caused by various gene mutations. Therefore, there remains an urgent need to develop safe and effective antitumor drugs. The antitumor effect of plant extracts, which are characterized by relatively low toxicity and adverse effect, has attracted significant attention. For example, increasing attention has been paid to the antitumor effects of tetramethylpyrazine (TMP), the active component of the Chinese medicine Chuanqiong, which can affect tumor cell proliferation, apoptosis, invasion, metastasis, and angiogenesis, as well as reverse chemotherapeutic resistance in neoplasms, thereby triggering antitumor effects. Moreover, TMP can be used in combination with chemotherapeutic agents to enhance their effects and reduce the side effect associated with chemotherapy. Herein, we review the antitumor effects of TMP to provide a theoretical basis and foundation for the further exploration of its underlying antitumor mechanisms and promoting its clinical application.

## 1 Introduction

Cancer remains a global public health threat caused by numerous factors, including aging, smoking (Bach, 2009; Thun et al., 2013), unhealthy dietary habits (Xiao et al., 2011), environmental pollution (Raaschou-Nielsen et al., 2013), lack of activity, and obesity (Wang et al., 2012; Islami et al., 2017). According to the global disease burden statistics published by the Washington University, cancer-related deaths account for approximately 15% of all deaths (Global Burden of Disease, Mortality and Causes of Death Collaborators, 2015). The Global Cancer Observatory 2020 database reported an estimated 19,292,789 cancer cases and 9,958,133 cancer-related deaths in 2020. Female breast cancer has surpassed lung cancer as the most common cancer, with an estimated 2,261,419 new cases, accounting for 11.7% of all cancer cases, followed by lung (11.4%), colorectal (10.0%), prostate (7.3%), and gastric cancers (5.6%). Lung cancer remained the leading cause of death due to cancer, with an estimated 1,796,144 deaths in 2020, accounting for 18.0% of all cancer-related deaths, followed by colorectal cancer (9.4%), liver cancer (8.3%), gastric cancer (7.7%), and female breast cancer (6.9%). The incidence of cancer and its associated mortality present significant variations according to region and sex (Sung et al., 2021). Compared with the 2020 estimates, new cases and deaths in 2040 are expected to increase by 49% and 62%, respectively. In the next 20 years, the burden of cancer will continue to increase (Cao et al., 2021).

**FIGURE 1:**
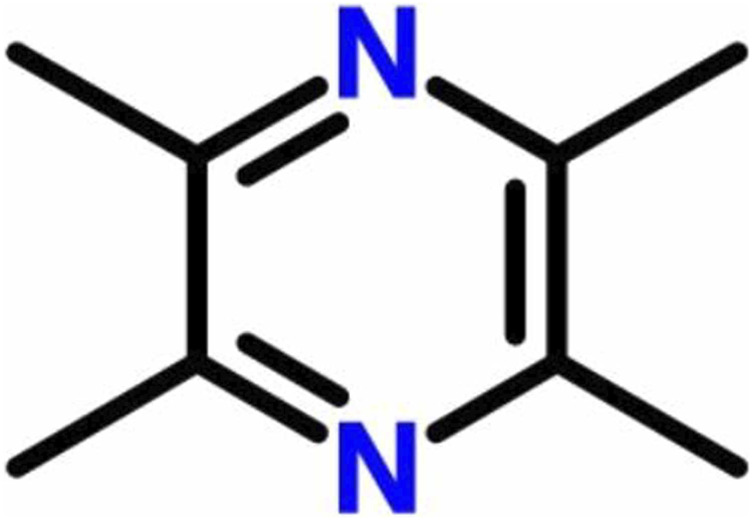
Chemical structure of tetramethylpyrazine.

The Chinese medicine Chuanqiong is the dry rhizome of the plant *Ligusticum chuanxiong* Hort, which belongs to the family *Apiaceae*. It has a pungent flavor with a unique aroma and is beneficial for the liver, gallbladder, and pericardium meridian. Chuanqiong has a high medicinal value with respect to activating blood circulation to dissipate blood stasis. The main components of Chuanqiong include alkaloids, phenols, and volatile oil (Wen et al., 2021). Among them, the main alkaloid component is tetramethylpyrazine (TMP) (Figure 1), also known as ligustrazine (chemical name: 2,3,5,6-tetramethylpyrazine; pyrazine; molecular formula: C_8_H_12_N_2_). TMP is an effective monomer obtained from the alkaloid of Chuanqiong and is also the main active substance in Chuanqiong. Previous studies have shown that TMP can exert antithrombosis, antiplatelet agglutination, antioxidation, and anti-ischemia reperfusion injury effects, improve microcirculation (Zou et al., 2018; Li et al., 2019; Zhou et al., 2020), and is widely used in various traumatic conditions and surgical treatments (Li et al., 2006; Yan et al., 2019). Additionally, TMP is used in the treatment of renal dysfunction (Sun et al., 2020), coronary heart disease (Wang et al., 2017), diabetes (Jiao et al., 2019), and cerebral infarction (Xu et al., 2017). At present, studies on the pharmacological role and clinical applications of TMP are very broad, and researchers are continuing to gain a deeper understanding of its characteristics.

In this review, we summarize the antitumor properties and potential mechanisms underlying the role of TMP in various tumors based on *in vitro* and *in vivo* studies. In addition to affecting the malignant biological behavior of tumor cells, TMP has protective effects on corresponding nontumor normal tissues. TMP can also be combined with chemotherapeutic agents to strengthen the damaging effects on tumors and can reduce the poisonous adverse effects caused by chemotherapeutic drugs, such as cardiac toxicity (Yang et al., 2019) and kidney toxicity (Michel and Menze, 2019). We compile these scientific pieces of evidence in this review to promote future research into the clinical application of TMP and further explore the potential treatment targets of TMP. We searched for almost all published papers related to the treatment of tumors with TMP in PubMed (https://pubmed.ncbi.nlm.nih.gov/) and summarized all the reports in this review. There is a large amount of research on TMP published in databases in Asia. Since these articles were not published in English, data provided in them remain unknown to researchers in other parts of the world.

## 2 Overview of tetramethylpyrazine

TMP has traditional significance in the treatment of ischemic cerebrovascular disease (Li et al., 2015) and in a wide range of other clinical applications. TMP, in combination with other drugs, is used widely in clinical practice. In recent years, studies have shown that TMP has various effects, including blood glucose and lipid regulation, hepatic protection, and mitigates inflammation and vascular endothelial cell injury (Guo et al., 2016), and has enormous potential for future clinical application. TMP is also widely used in basic clinical treatment, and researchers have very broadly studied various aspects of it, such as its sedative and analgesic effects (Liang et al., 2005; Gao et al., 2008), antithrombotic effects (Cai et al., 2014), protection against ischemia reperfusion injury (Qian et al., 2014; Zhang et al., 2014; Zhang et al., 2018), and effects on blood vessels (Shan Au et al., 2003; Xu et al., 2014).

## 3 Antitumor mechanism underlying the effects of tetramethylpyrazine in multiple organs

**FIGURE 2:**
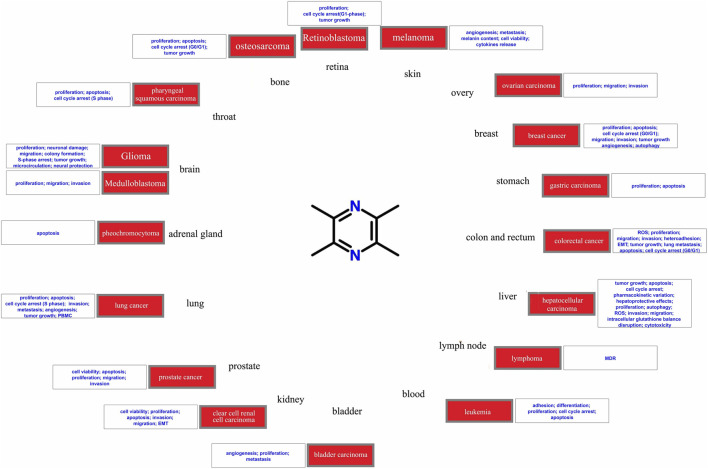
Diseases involved in the current study of the antitumor potential of tetramethylpyrazine.

TMP has shown antitumor effects on various tumor types (Figure 2), including brain glioma (Cai et al., 2014), breast (Fan et al., 2021), prostate (Zhou et al., 2020), and lung cancers (Huang et al., 2018). Tumor growth can be reversed by applying different concentrations of TMP to tumor cells. Researchers have developed *in vitro* strategies for assessing the dosage, efficacy, and potential molecular mechanisms underlying the effects of TMP in different tumor cells (Fu et al., 2008; Huang et al., 2018; Zhou et al., 2020). TMP exhibits various antitumor effect on different tumor cells. TMP regulates various molecular signal pathways to alter the malignant biological behavior of tumor cells, including proliferation (Fu et al., 2008), cell cycle regulation (Yu et al., 2012), apoptosis (Cheng et al., 2007), invasion (Xu et al., 2018), metastasis (Fu et al., 2008), and angiogenesis (Jia et al., 2016). TMP also exhibits antiproliferative and antiangiogenic potential in a variety of *in vivo* models including a xenograft mouse model (Yu et al., 2012) and other experimental animal models (Fu et al., 2008). Overall, TMP has shown promising potential as an antitumor drug. In this review, we elaborate on the resistance of tumors to TMP according to body systems and the significant protection of organs around the tumor.

### 3.1 Brain and nervous system

Glioma is the most common tumor of the central nervous system that originates in glial cells. Over the past 30 years, the incidence of glioma has increased at a rate of 1%–2% per year (Chen et al., 2020). At present, treatment modalities available for glioma include surgery, chemotherapy, and radiotherapy; gene therapy and immunotherapy have also been actively studied. However, the overall efficacy is not ideal (Eslahi et al., 2021). Fu et al. (2008) investigated the possible therapeutic efficacy of TMP against a rat glioma cell line (C6) and gliomas transplanted into rat brains. The authors found that TMP can suppress glioma activity, including growth, and protect neurons against glioma-induced excitotoxicity. C-X-C chemokine receptor type 4 (CXCR4), initially discovered for its involvement in human immunodeficiency virus entry and leukocyte trafficking, is overexpressed in more than 23 human cancers. Zhuang et al. have reported that TMP could downregulate CXCR4 in C6 cells, and further inhibit cell migration, proliferation, and colony formation, and induce S phase arrest more effectively than AMD3100 (a CXCR4 antagonist). The *in vivo* experiments in rats implanted with C6 cells showed results analogous to those found *in vitro* (Yu et al., 2012). In subsequent studies, TMP was found to effectively promote cerebral neurocyte survival by inhibiting hydrogen peroxide (H_2_O_2_)-induced increase in the intracellular concentration of Ca^2+^ and glutamate release. Upon using a glioma–neuronal coculturing system, TMP was more effective in these functions compared with AMD3100 (Chen et al., 2013). In 2014, Zhuang et al. studied the presence of high levels of invasion caused by neovascularization of gliomas using a venous endothelial cell line (ECV304) and a rat model. TMP inhibited neovascularization, fibrosis, and thrombosis under pathological conditions, contributing to the downregulation of CXCR4 through the SDF-1/CXCR4 axis (Cai et al., 2014).

There is a relative paucity of research on other tumors of the nervous system. The classic PI3K/AKT pathway remains to be fully investigated, and TMP has been found to affect the downstream functional protein, resulting in a change in tumor biological behavior. In a medulloblastoma cell line (Daoy), TMP inhibited the PI3K/AKT and mTOR signaling pathways by upregulating miR-211 to affect the proliferative, migratory, and invasive abilities of Daoy cells with the increase in the expression of caspase-3, caspase-9, and Bax, and a decrease in the expression of Bcl-2, MMP-2, MMP-9, and Vim (Xu et al., 2018). TMP blocked H_2_O_2_-induced apoptosis by regulating the expression of the members of the Bcl-2 family, suppressing cytochrome c release, and activating the caspase cascade in the rat pheochromocytoma-derived cell line PC12 (Cheng et al., 2007).

In contrast to the negative impact on tumor tissue, TMP plays a protective role in brain damage via a mechanism mainly related to the antioxidation or antiapoptotic pathways. *In vivo* and *in vitro* experimental studies have shown that TMP can improve cobalt chloride-induced oxidative stress and brain nerve injury, via a mechanism involving the increased expressions of nuclear factor erythroid 2-related factor 2 (NRF2) and glutamyl cystine ligase to promote the synthesis of glutathione (GSH) and reduce the levels of reactive oxygen species (ROS). Meanwhile, TMP inhibited the expressions of hypoxia-inducible factor 1α (HIF-1α) and nicotinamide adenine dinucleotide phosphate oxidase 2 (NOX2), to inhibit ROS production mediated by HIF-1α and NOX2. Through these two antioxidant pathways, TMP blocks apoptosis, restores mitochondrial function, and protects the brain cells (Guan et al., 2015). TMP may stimulate neuronal differentiation of human neuroblastoma SH-SY5Y cells by enhancing the recruitment of Ac-H3 and Ac-H4 to the topoisomerase II β gene promoter region to regulate neuronal development (Yan et al., 2014). TMP can also activate the mitogen-activated protein kinase (MAPK) signaling pathway by promoting the phosphorylation of extracellular signal-regulated kinase (ERK)1/2 and reducing the phosphorylation of P38, thereby promoting brain neural stem cell proliferation and differentiation into neuronal cells under hypoxic conditions (Tian et al., 2010). TMP has a therapeutic effect on neurological diseases. TMP effectively reversed scopolamine-induced memory impairment in rats by improving postsynaptic protein synthesis and restoring the signal conduction of cyclophosphate/protein kinase A/the cAMP response element-binding protein (CREB) pathway (Wu et al., 2013). In cultured microglial cells stimulated with Aβ25-35, TMP repressed the inflammatory response in the presence of interferon (IFN)-γ and blocked ROS generation and phosphorylation of Akt to alleviate the inflammatory progression of Alzheimer’s disease (Kim et al., 2014).

### 3.2 Respiratory system

Lung cancer has the highest incidence and mortality rate worldwide. Among its subtypes, nonsmall cell lung cancer (NSCLC) is the main histological type and accounts for 85% of all cases. Most patients are already in the middle and late stages of the disease when diagnosed (Herbst et al., 2018). In recent years, precision treatment modalities for lung cancer, such as targeted therapy, antiangiogenic treatment, and immunotherapy, have been rapidly developed. However, the survival period of lung cancer is still unsatisfactory (Hirsch et al., 2017). A study on the lung cancer cell lines A549 and 95D conducted by Huang et al. (2018) showed that TMP decreased cell viability in a dose- and time-dependent manner and suppressed the carcinogenesis of lung cancer cells by arresting the cell cycle at the S phase and inducing mitochondria-dependent apoptosis by regulating caspase-3 and Bax/Bcl-2. Cyclooxygenase (COX)-2 plays an important role in tumorigenesis and is a critical factor for the invasion and metastasis of lung cancer. Zheng et al. (2012) found that TMP exhibited the same dose- and time-dependent inhibitory effect on the proliferation of the lung cancer cell line A549 by suppressing cell cycle progression, which resulted in the inhibition of invasion *in vitro* and suppression of metastatic growth in an *in vivo* metastatic nude mouse model by targeting COX-2.

Hematogenous metastasis is often diagnosed in early-stage lung cancer, particularly, small cell lung cancer. Tumor growth and metastasis of lung cancer depend on the formation of a neovasculature. Antiangiogenic treatment has gradually attracted significant attention (Yi et al., 2019). Research on the pulmonary vascular cell model (microvascular endothelial cell line: HMEC-1) showed that TMP could suppress angiogenesis and tumor growth in lung cancer by blocking the BMP/Smad/Id-1 signaling pathway in a dose- and time-dependent manner. In addition, the administration of TMP inhibited the tumor growth of A549 xenografts in nude mice, with reduced expression of CD31, phosphorylated Smad1/5/8, and Id-1 (Jia et al., 2016). Lung cancer patients predominantly express type 2 cytokines. After investigating peripheral blood mononuclear cells obtained from lung cancer patients, Wei et al. (2002, 2004) reported that TMP could reverse the predominant type 2 status. This predominant expression of Th2 type cytokines may be related to a lower expression of T-bet or a higher expression of GATA3, with which TMP interferes.

The interaction of platelets in the blood and with TMP can also affect lung cancer progression. TMP, as a blood-activating agent, could increase the adhesion of the lung cancer cell lines PGCL3 and PAa to fibronectin but inhibit the invasion of PGCL3 cells in a Boyden chamber (Zhang et al., 1999). In a comparison of advanced cases of lung carcinoma and matched control subjects, TMP has been shown to have an antimetastatic effect on lung carcinoma as it inhibited the adhesion and aggregatory functions of blood platelets and the activity of coagulation factors (Chen et al., 1997).

### 3.3 Urinary system

Prostate cancer is a common malignant tumor among men, often treated with antiandrogen therapy. Most patients will be nondependent on androgen after 18–24 months of treatment (Cornford et al., 2021).

In prostate cancer cells, TMP treatment reduces viability and increases the rate of apoptosis in a dose-dependent manner. During the expression of long noncoding RNA, DPP10-AS1 is upregulated and becomes associated with CREB-binding protein to induce H3K27ac enrichment at the promoter region of the forkhead box M1 (*FOXM1*) gene (Zhou et al., 2020). Hormone-refractory prostate cancer (HRPC) is the final progression stage of prostate cancer and the most difficult to treat. Most patients have concomitant bone metastasis or pelvic lymph node metastasis when the diagnosis is confirmed. Whole-body chemotherapy or palliation is the main treatment modality for HRPC; however, the clinical efficacy is not ideal (Heber et al., 2019). In the HRPC cell line PC-3, TMP reduces cell proliferation and promotes apoptosis by modulating the availability of eIF4E mainly through the mTOR and MEK/ERK signaling pathways to inhibit cap-dependent translation (Han et al., 2015). In addition, Zhou et al. (2017) reported that TMP inhibited PC-3 cell proliferation, migration, and invasion by downregulating *FOXM1* through treatment with TMP and a pcDNA–FOXM1 plasmid.

Clear cell renal cell carcinoma (ccRCC) is the commonest type of kidney cancer. The associated mortality rate is as high as 47%, making it a significant threat to patients (Jonasch et al., 2021). The antitumor efficacy of TMP was investigated in the human ccRCC cell line and found to inhibit ccRCC cell viability, proliferation, apoptosis, invasion, and migration by inhibiting the NKG2D-related signaling pathway to further suppress epithelial–mesenchymal transition (EMT) progression. The binding of NKG2D to its ligands activates natural killer (NK) cells to increase NK cell-mediated cytotoxicity against cancer cells with a high expression of major histocompatibility complex class I chain-related molecules A and B (Luan et al., 2016). BA-12, a TMP–betulinic acid derivative, exhibits potent antitumor activities by blocking angiogenesis to inhibit the growth and metastasis of bladder carcinoma cells (T24); this involves interfering with GSH metabolism and activating glycerophospholipid metabolism (Cui et al., 2019; Cui et al., 2020).

TMP plays a renoprotective role by protecting kidney tissues and cells against apoptosis, has an antioxidant function to ameliorate kidney injury and kidney tissue fibrosis. Yang et al. (2011) found that TMP can improve the pathological conditions of rats with diabetic nephropathy induced by streptozocin by decreasing the expression of vascular endothelial growth factor (VEGF). Another study reported that TMP plays a protective role against kidney injury among rats. Its mechanism may involve the inhibition of the P38 MAPK expression and the transcriptional factor forkhead box O1 which blocks the signal transduction pathway mediated by these two proteins, thus playing an antiapoptotic role. In addition, TMP plays a protective role against human renal proximal tubule injury induced by sodium arsenate, via a mechanism involving the inhibition of ROS production, increasing the level of GSH, and increasing cytochrome C oxidase activity to improve mitochondrial dysfunction (Gong et al., 2015). TMP also induces tubulointerstitial fibrosis and resists ureteric obstruction via multiple pathways. Possible mechanisms involved in this change include the transdifferentiation of renal tubular mesenchymal cells and exerting antioxidant effects. This process downregulates the expression of TGF43L protein and connective tissue growth factor, and upregulates hepatocyte growth factor and BMP-7 (Yuan et al., 2012).

### 3.4 Blood and immune system

Acute leukemia is the commonest malignant tumor among children and adolescents, mainly treated with chemotherapy. Treatment failure and recurrence caused by reduced chemotherapy sensitivity and drug resistance are major challenges faced during leukemia treatment (Cartel et al., 2021). Lymphocyte function-associated antigen-1 (LFA-1) is expressed on the surfaces of the T-cell leukemia cell line SKW-3. Red blood cells coupled with intercellular adhesion molecule-1 (ICAM-1) are carriers for LFA-1 and ICAM-1, which mediate the adhesion properties of cells (Zhao et al., 2000). Small doses of TMP induce the nonterminal differentiation and proliferation of the leukemia cell line HL-60 in a dose- and time-dependent manner, in addition to synergistically blocking the cell cycle progression of HL-60 cells in the G0/G1 phase (Wu et al., 2011). In acute lymphoblastic leukemia cell lines Jurkat and SUP-B15, TMP induces apoptosis and causes cell cycle arrest at the G0/G1 phase by downregulating GSK-3β, which further prevents the induced translocation of NF-κB and c-myc from the cytoplasm to the nucleus (Wang et al., 2015). In the leukemia cell line U937, TMP inhibits cellular proliferation and induces apoptosis, via a mechanism possibly associated with its impact on the cell cycle distribution, regulation of Bcl-2 expression, and finally via caspase-3 activation (Wang et al., 2015).

Research has shown that TMP also plays an effective role in the treatment of lymphoma. Among 60 patients, TMP was found to act as a salvage agent in combination with chemotherapy and could increase the response rate, prolong the progression-free survival with manageable toxicity, and correlate with p-glycoprotein (P-gp) expression in relapsed or refractory non-Hodgkin’s lymphoma (NHL) (Yang et al., 2010).

### 3.5 Digestive system

Hepatocellular carcinoma (HCC) is one of the most common malignant tumors. The latest data show that the incidence of HCC is the sixth highest, with the third highest mortality rate (Yeung et al., 2022) among all malignant tumors. More than 80% of the HCC patients are already in an advanced stage at diagnosis or lose the opportunity for surgery. Even among patients who have undergone radical surgery, the 2-year recurrence rate remains at up to 50% (Shiffman, 2018). This high recurrence rate may be related to the resistance to single-agent chemotherapy or combined chemotherapy (Perboni et al., 2010). TMP could significantly inhibit tumor development in rats with diethylnitrosamine-induced HCC by inducing apoptosis and cell cycle arrest at the G2/M phase through the mitochondrial apoptotic pathway (Cao et al., 2015). Upon detecting pharmacokinetic variations of TMP phosphate after oral administration in mice with hepatic precancerous lesions, TMP was partly effective in protecting the liver from carcinogenesis initiated by diethylnitrosamine; hepatic insufficiency could alter its pharmacokinetics (Feng et al., 2014).

TMP significantly inhibited HCC cell line (HepG2) proliferation, and induced cell cycle arrest at the G0/G1 checkpoint and caspase-dependent mitochondrial apoptosis *in vitro* (Bi et al., 2016). TMP induced ROS generation and the inhibition of ROS reduced the antitumor function to regulate autophagy and proliferation by cleaving caspase-3 and PARP in HepG2 cells and xenograft tumor models (Cao et al., 2015). As a major active component of TOGA, a novel conjugate, TMP could prevent the invasion and migration of HepG2 cells induced by tumor-associated macrophages or IL-1β through the IL-1R1/IκB/IKK/NF-κB signaling pathway with the decreased expression of the EMT-related proteins Snail and Vimentin (Wang et al., 2020). As a model compound, 3-hydro-2,2,5,6-TMP (DHP-3) exerted cytotoxic activity by disrupting the intracellular GSH balance in HepG2 cells in a concentration range of 10 µM–1 mM and significantly so at the highest concentration (Ishida et al., 2012). TMP could alleviate the hepatotoxicity resulting from cyclophosphamide treatment as evidenced by improvement in the structure and function of the liver, and inhibition of oxidative stress and inflammation with accompanying pyroptosis, which was positively correlated with the inhibition of the Txnip/Trx/NF-κB pathway (Ma et al., 2021).

TMP also has an inhibitory effect against gastrointestinal tumors. The induction of apoptosis by TMP in the gastric carcinoma cell line SGC7901 is associated with the activation of the ROS/AMPK (AMP-activated protein kinase) pathway, and AMPK activation induces apoptosis through the mitochondrial apoptotic pathway (Yi et al., 2013). TMP also exhibits significant antiproliferative and proapoptotic properties, regulated by NF-κB, p65, cyclinD1, and p16 in SGC-7901 cells (Ji et al., 2014). TMP could induce colorectal cancer cell line (SW480 and CT26) apoptosis via a p53-dependent mitochondrial pathway and cell cycle arrest at the G0/G1 phase; TMP-induced apoptosis and cell cycle arrest were markedly reversed by pifithrin-α (a p53 inhibitor) (Bian et al., 2021). TMP possesses a water-soluble pyrazine skeleton and can inhibit the proliferation and metastasis of cancer cells. Zou et al. (2018) synthesized a compound by replacing the trimethoxyphenyl group of piperlongumine 1 with a TMP moiety. In the colorectal cancer cell line HCT-116, this compound increased ROS levels, and inhibited proliferation, migration, invasion, and heteroadhesion to a greater extent than piperlongumine 1 through EMT induced by TGF-β1 and Wnt/β-catenin activation by inhibiting Akt and GSK-3β phosphorylation, in addition to suppressing tumor growth and lung metastasis *in vivo* to prolong the survival of tumor-bearing mice (Zou et al., 2018).

### 3.6 Reproductive system

Breast cancer is one of the most common malignant tumors worldwide and its associated mortality rate is the second highest among all malignant tumors (Siegel et al., 2020).

Based on the expression of estrogen receptor (ER), progesterone receptor (PR), human epidermal growth factor receptor-2 (HER2), and Ki-67, breast cancer can be divided into the following types: the luminal (luminal or hormone receptor positive) A, luminal B, Her-2 overexpression, and basal-like (substrate) types (Viale et al., 2019). The molecular profiles of breast cancer are closely related to their pathological features and clinical prognosis (Tran and Bedard, 2011). Endocrine treatments, such as tamoxifen administration and chemotherapy often cause drug resistance, which leads to a relatively poor prognosis (Viedma-Rodríguez et al., 2014). In the breast cancer cell line MDA-MB-231, TMP inhibited cell survival, and induced apoptosis and cell cycle arrest at the G0/G1 phase. The *in vivo* findings among xenograft tumors established in nude mice were consistent with those found *in vitro* (Pan et al., 2015). TMP significantly inhibited the viability, migration, and invasion rates, and increased the apoptosis of cells in a dose-dependent manner, as in MDA-MB-231 cells, by inhibiting the activity of Akt and increasing the activity of caspase-3 (Shen et al., 2018). Signal transducer and activator of transcription 3 (STAT3) is overexpressed and hyperactivated in tumors. Statmp-151, a TMP derivative, could also act as a novel small molecule Stat3 inhibitor against breast cancer by influencing the mitochondrial membrane potential and ROS generation (Fan et al., 2021).

ER/PR-positive patients account for approximately 70% of all breast cancer patients; triple-negative breast cancer accounts for 19%, and the Her-2 overexpression type accounts for the remainder (Viale et al., 2019). ER positivity is often considered a good prognostic factor. Due to the lack of corresponding treatment targets, triple-negative breast cancer is considered to have the worst prognosis among patients with breast cancer. TMP, as a component of SANT, a novel Chinese herbal monomer combination, decreased tumor growth and angiogenesis *in vivo* and *in vitro* by modulating autophagy in heparinase-overexpressed triple-negative breast cancer. During this process, the expression levels of the *ATG16L1*, *ATG9B*, and *ATG4D* genes increased and those of the *TMEM74* and *TNF* genes decreased. Additionally, the protein levels of HB-EGF, thrombospondin-2, amphiregulin, leptin, IGFBP-9, EGF, coagulation factor III, and MMP-9 (pro and active forms) in the tumor decreased, whereas those of serpin E1 and platelet factor 4 increased (Li et al., 2021).

Chemotherapy is an important strategy for treating breast cancer; however, the occurrence of drug resistance during chemotherapy often leads to failure of breast cancer treatment (Holohan et al., 2013). Drug resistance has become one of the main obstacles to breast cancer treatment. Consensus regarding the use of TMP to relieve tumor resistance has been reached.

Due to a lack of an effective early detection method, typical clinical symptoms are the most important reasons for delaying ovarian cancer diagnosis. Approximately 70% of the patients with ovarian cancer are diagnosed at an advanced stage (Snijders et al., 2017). The high mortality rate of ovarian cancer is attributed to the characteristics of its distant metastasis (Li et al., 2005). TMP can reduce the viability, proliferation, migration, and invasion ability of the human ovarian carcinoma cell lines SK-OV-3 and OVCAR-3 by regulating miR-211, and the EMT is also involved in this process (Zhang et al., 2021). TMP also inhibits the invasion and migration of SK-OV-3 cells by decreasing the expression of IL-8 through the ERK1/2, p38, and AP-1 signaling pathways, and IL-8 expression was significantly inhibited after coincubation with PD98059 (an ERK inhibitor) and SB203580 (a p38 inhibitor) (Yin et al., 2011).

### 3.7 Other organs

Malignant melanoma has a high invasiveness and is associated with a high mortality rate. Early-stage melanoma can be surgically resected, and the 5-year survival rate can reach 95%. However, advanced, or unresectable melanoma constitutes a major challenge in the treatment of melanoma (Ubellacker et al., 2020). In the melanoma cell line B16F10 spontaneous metastasis model, TMP inhibited tumor metastasis through its antiangiogenic activity by decreasing the expression of CD34 and VEGF in the primary tumor tissue and reducing the number of metastase nodi on the lung surface (Chen et al., 2009). TMP was also studied in an ultraviolet A-induced melanoma/keratinocyte coculture system, in which it regulates melanogenesis by enhancing inflammation and decreasing the levels of melanogenic factors (TRP1, MITF, and MAPK) (Yeom et al., 2014).

Retinoblastoma is the most common ocular tumor among children and causes extensive damage. Wu et al. (2017) reported interesting findings on the treatment of retinoblastoma with TMP. In the retinoblastoma cell line WERI-Rb1, TMP significantly downregulated the expression of CXCR4 in a time-dependent manner by reducing the expression of the transcription factor nuclear respiratory factor-1 (Nrf-1). Moreover, it inhibited cell proliferation as an effective CXCR4 antagonist (such as AMD3100) and induced G1-phase arrest in cells seeded at high-density. In addition, TMP protected normal retinal neurocytes from H_2_O_2_-induced damage by downregulating CXCR4 (Wu et al., 2017; Wu et al., 2019).

In osteosarcoma cell lines (MG-63, SAOS-2, and U2OS), TMP inhibited cell proliferation and induced apoptosis and G0/G1 arrest in a dose-dependent manner by upregulating the expressions of cytosolic NF-κB and p65, while downregulating the nuclear expressions of NF-κB, p65, Bcl-2, and cyclin D1. In addition, TMP exerted an antitumor effect against osteosarcoma in a xenograft tumor mouse model and exhibited a low level of toxicity (Wang et al., 2013). Wang et al. (2019). Synthesized TMP dimers and seven TMP tetramers were linked by alkane diamine, most of which showed better cytotoxicity than the TMP monomer. The TMP dimer 8e linked with decane-1,10-diamine exhibited the highest cytotoxicity in the pharyngeal squamous carcinoma cell line FADU and induced the apoptosis of FaDu cells by depolarizing the mitochondrial membrane potential and S phase cell cycle arrest.

## 4 Effects and reverse function of multidrug resistance to chemotherapeutic agents

Tumor multidrug resistance (MDR) is a major obstacle to cancer chemotherapy. MDR involves the imbalance of multiple mechanisms, including the decrease in concentration of intracellular drugs, changes in drug target molecules, metabolic detoxification, and DNA damage repair function (Tinoush et al., 2020). The need to overcome MDR in tumor cells and improve the efficacy of antitumor drugs remains a key challenge that must be addressed. Recent studies suggest that TMP may reverse tumor MDR, although the mechanism for this remains elusive.

Chemotherapy significantly impacts the physique of the patient, which reduces their tolerance of chemotherapy and overall quality of life. Therefore, it is necessary to continue to explore new treatment options to allow patients undergoing chemotherapy to obtain more benefits. TMP treatment of patients with advanced tumors can reduce toxicity and enhance the efficacy of chemotherapy. For patients with poor general conditions and a strong willingness to use traditional Chinese medicine, a combination of traditional Chinese medicine and chemotherapy may be an option. The amphiphilic paclitaxel-ss-TMP conjugate that readily self-assembles into stable nanoparticles in aqueous solution belongs to a redox-responsive carrier-free nanosystem with intrinsic amphiphilicity, which exhibits higher cytotoxicity by being associated with a greater apoptosis rate and cell cycle arrest than monotherapy or combination therapy with free drugs. In addition, this treatment also shows tumor-specific accumulation and excellent antitumor activity in A2780 xenograft mice (Zou et al., 2021).

### 4.1 Adriamycin

Adriamycin, also known as doxorubicin, is an antitumor antibiotic that inhibits the synthesis of RNA and DNA. This drug nonspecifically targets the cell cycle and has a damaging effect on tumor cells in various phases of the growth cycle. Therefore, it exhibits a wide range of biochemical effects on the body and has a strong cytotoxic effect (Li et al., 2021; Riaz and Hussain, 2021). The effectiveness of Adriamycin in the treatment of cancer has been limited by the development of drug resistance. TMP showed a potentiating effect on the cytotoxicity of Adriamycin *in vitro* and partly reversed adriamycin resistance in the resistant mouse cell line EAC (Hu et al., 1993). TMP can reverse MDR of the HCC cell line HepG2/ADM by enhancing the density of adriamycin in the cell and increasing its cytotoxicity. In this process, the transcriptional activity of MDR1 and the expression of P-gp170 decreases (Mei et al., 2004). TMP also reverses MDR in the HCC cell line BEL-7402/ADM (Wang et al., 2010). Zhou (2007) showed that the average initial adriamycin efflux rate in the breast cancer cell line MCF-7/ADR was higher than that in MCF-7. After treatment with TMP, the drug efflux rate of the MCF-7/ADR cells was reduced to approximately half of that in cells without inhibitors. In the breast cancer cell line MCF-7/Dox, TMP increased the intracellular concentration of adriamycin and inhibited the P-gp-mediated efflux of doxorubicin in a dose-dependent manner, which was induced by inhibiting the ATPase activity of P-gp (Zhang et al., 2012). DLJ14, a TMP piperazine derivative, could serve as a promising chemo-sensitization candidate for the reversal of MDR in the breast cancer cell line (MCF-7/A). It increases the intracellular accumulation of adriamycin by inhibiting the GSH level and the GSH peroxidase and GSH S-transferase (GST) activity (Zhang et al., 2014). Combination treatment with DLJ14 and adriamycin could inhibit the growth of adriamycin-resistant MCF-7/A cells by inhibiting the EGFR/PI3K/Akt survival pathway and inducing apoptosis via the mitochondrial-apoptosis pathway (Chen et al., 2014). Overexpression of GSTπ is one of the mechanisms that contributes to MDR. DLJ14 may trigger the reversal of MDR in adriamycin-resistant human myelogenous leukemia (K562/A02) cells by modulating the expression of GSTπ and GST-related enzymes (Song et al., 2011). The cardiotoxicity and endotheliotoxicity of adriamycin limit its clinical application in cancer treatment. TMP protects the vascular endothelium against adriamycin-induced injury by upregulating 14-3-3*γ* expression, promoting the translocation of Bcl-2 into the mitochondria, closing mPTP, maintaining MMP, inhibiting the RIRR mechanism, suppressing oxidative stress, improving mitochondrial function, and alleviating adriamycin-induced endotheliotoxicity (Yang et al., 2019). Epirubicin is an isomer of adriamycin. TMP reverted epirubicin resistance by inhibiting the JAK2/STAT3 pathway and decreasing fibrinogen gamma chain (FGG) expression in breast cancer; the elimination of cancer stem cells has also been observed during this process (Liu et al., 2020).

### 4.2 Cisplatin

Cisplatin inhibits DNA function and cell mitosis (Hamano et al., 2021). Cisplatin has a wide antitumor spectrum and is used in the treatment of head and neck squamous cell carcinoma, ovarian cancer, embryonic cancer, lung cancer, and thyroid cancer (Fang et al., 2021; Prayuenyong et al., 2021). In Lewis lung cancer mice, TMP with cisplatin exhibited additional or synergistic effects with respect to inhibiting tumor growth effectively; the mechanism involved reducing the expression of the angiogenesis-promoting factor VEGF and increasing the expression of the angiogenesis inhibitors KLF4 and ADAMTS1 (Tang et al., 2017). Ai et al. (2016)-designed TMP–curcumin hybrids (10a–u). Compound 10d inhibited the proliferation of the drug-sensitive lung cancer cell lines A549, SPC-A-1, and LTEP-G-2, and drug-resistant A549/DDP cells, by suppressing the TrxR/Trx system and promoting intracellular ROS accumulation and cancer cell apoptosis. Compound 10d also inhibited the growth of implanted human drug-resistant lung cancer in mice. TMP enhances the cytotoxic effect of antitumor agents on the bladder cancer cell lines Pumc-91/ADM and T24/DDP in response to adriamycin, which results in cell cycle arrest in the G1/S phase due to the decrease in the levels of MRP1, GST, and Bcl-2 and increase in the levels of topoisomerase-II (Wang et al., 2016). Cisplatin is one of the most effective broad-spectrum cancer chemotherapy drugs that exhibits serious adverse effects, such as acute renal injury. A study on cisplatin-treated rats showed that TMP might be a potential candidate for neoadjuvant chemotherapy due to its antioxidant, antiinflammatory, and antiapoptotic effects, in addition to its effect on Nrf2, the HMGB1/TLR4/NF-κB signaling pathway, and PPAR-γ expression (Michel and Menze, 2019).

### 4.3 Paclitaxel

Paclitaxel, a natural secondary metabolite obtained from the bark of *Taxus chinensis* var. *mairei* via cell culture exhibits a good antitumor effect, particularly against ovarian (Lau et al., 2020), cervical (Kitagawa et al., 2015), and breast cancers (Untch et al., 2016). For the treatment of NSCLC, dequalinium-modified paclitaxel plus TMP micelles destroyed vasculogenic mimicry channels and inhibit tumor metastasis (Xie et al., 2019). In the ovarian cancer cell lines A2780 and SKOV3, TMP in combination with paclitaxel suppressed angiogenesis by inhibiting the ERK1/2 and Akt pathways and promoted the apoptosis of tumor cells to enhance the antitumor effects of paclitaxel compared with treatment alone. In A2780 xenograft mouse models, TMP augmented the antitumor effects of paclitaxel by influencing cell proliferation and angiogenesis as well as decreasing paclitaxel toxicity (Zou et al., 2019).

## 5 Derivatives and compounds of tetramethylpyrazine

Many active monomers used in traditional Chinese medicine and their derivatives or analogs can be used to overcome the low bioavailability of traditional Chinese medicines. They can ameliorate the clinical symptoms and prevent tumor recurrence. In addition, traditional Chinese medicine compounds can strengthen the antitumor effects (compared with single Chinese medicine monomers) (Luan et al., 2020). Below, we summarize the characteristics of three TMP derivatives and compounds [T-OA, DT-010, and (E)-2-(2-chlorostyryl)-3,5,6-trimethylpyrazine (CSTMP)] and expound their role in tumor treatment.

### 5.1 T-OA

T-OA, an antitumor TMP derivative with the chemical name 3βhydroxyolea-12-en-28-oic acid-3,5,6-trimethylpyrazin-2-methylester, has been shown to have effective anticancer activity (Fu et al., 2017). It exerts its antitumor activity and pharmacokinetic characteristics by preventing the expression of the nuclear transcription factor NF-κB/p65 and COX-2 in S180 mice (sarcoma) (Wang et al., 2013). Compared with cisplatin, T-OA is more toxic to the HCC cell line Bel-7402 than to three other cancer cell lines (HeLa, HT-29, and BGC-823), and plays a role in apoptosis by preventing the expression of NF-κB/p65 and COX-2 in Bel-7402 cells (Zhang et al., 2015). Although the poor solubility of T-OA results in low oral bioavailability, T-OA liposomes could significantly promote its intestinal lymphatic transport and enhance its oral bioavailability (Li et al., 2020). T-OA(6a), as a T-OA derivative designed by Chu et al. (2014), induced apoptosis in the HCC cell line HepG2 via nuclear fragmentation and exhibited lower nephrotoxicity. Additionally, T-OA(6a) exhibited good levels of cytotoxicity and possessed better hydrophilicity than T-OA in the cancer cell lines HepG2, HT-29, HeLa, and BGC-823 (Chu et al., 2014).

### 5.2 DT-010

DT-010, a novel synthetic compound of Danshensu and TMP, has been found to possess a cardioprotective effect with respect to myocardial ischemia/reperfusion injury in clinical studies (Xie et al., 2021). In the breast cell line MCF-7, DT-010 was more potent than TMP, Danshensu, or a combination of the two with respect to potentiating doxorubicin-induced toxicity; cotreatment with DT-010 and doxorubicin increased the level of apoptosis relative to doxorubicin alone. DT-010 and doxorubicin exhibit a synergistic antitumor effect in breast cancer by downregulating the glycolytic pathway and GRP78. DT-010 also protects against doxorubicin-induced cardiotoxicity (Wang et al., 2016), restores doxorubicin-induced apoptosis, and significantly inhibits growth when combined with doxorubicin. DT-010 overcomes doxorubicin resistance through a dual action by simultaneously inhibiting P-gp-mediated drug efflux and influencing the metabolic process (Zhou et al., 2019). DT-010 potently inhibits cell proliferation by inducing cytotoxicity and promoting cell cycle arrest in the breast cancer cell lines MCF-7 and MDA-MB-231; this effect is attributed to the suppression of the mitochondrial function. Further studies have shown that DT-010 suppresses succinate-induced mitochondrial respiration and impairs mitochondrial complex II enzyme activity to trigger ROS generation and mitochondrial dysfunction (Wang et al., 2016). Tang et al. (2018) suggested DT-010 as a potential therapeutic agent for effectively combating the cardiotoxicity of doxorubicin. DT-010 prevents doxorubicin-induced morphological changes and directly inhibits the generation of ROS. As cardiotoxicity is multifactorial, DT-010 could also inhibit the induction of autophagosome formation by regulating the upstream Akt/AMPK/mTOR signaling pathway (Tang et al., 2018).

### 5.3 CSTMP

CSTMP, a TMP analog, was designed and synthesized based on the pharmacophores of TMP and resveratrol (Ding et al., 2016). In the lung cancer cell line A549, CSTMP inhibited cell proliferation and induced cell cycle arrest and apoptosis through IRE1α-TRAF2-ASK1 complex-mediated ER stress, JNK activation, and mitochondrial dysfunction. The process could also be reversed by treatment with IRE1α siRNA (Zhang et al., 2016). CSTMP showed significant cytotoxic effects in the human myeloma cell line RPMI8226 by promoting caspase- and mitochondria-dependent apoptosis with increasing expressions of endoplasmic reticulum stress-related proteins (CHOP, GRP78, GRP94, and cleaved caspase-12) and activation of multiple ER stress transducers (PERK-eIF2α, IRE1α, and ATF6) (Sun et al., 2016).

Certain synthesized derivatives or compounds exhibit superior anticancer effects and fewer adverse effects than the available chemotherapeutic drugs or TMP monomers. These advantages are attributed to their relatively improved biosafety and lower toxicity, and successfully targeting proliferation, apoptosis, invasion, angiogenesis, cell cycle, and mitochondrial membrane potential. Synthetic TMP–betulin derivatives (TBs) have been proposed, with most demonstrating a better antitumor activity than betulin in HeLa, HepG2, BGC-823, and HT-29 cell lines. Among them, compound TB-01 showed the best antitumor effect and the lowest toxicity on normal cells, and demonstrated better cytotoxicity than cisplatin toward cancer cells. In addition, TB-01 induced early apoptosis in HepG2 cells and blocked the cell cycle at the G1 phase (Guo et al., 2020). Xu et al. (2015) designed novel TMP–triterpene derivatives, among which compound 4a exhibited better cytotoxic activity against Bel-7402, HT-29, MCF-7, HeLa, and HepG2 than cisplatin by depolarizing the mitochondrial membrane potential and increasing the intracellular free Ca^2+^ concentration (Xu et al., 2015). Dipeptide derivatives of TBA (a TMP compound) and amino acid were also designed by Xu et al. (2017), of which BA-25 exhibited the highest cytotoxic activity in tumor cell lines (HepG2, HT-29, HeLa, BCG-823, and A549) compared with cisplatin. BA-25 induced apoptosis associated with loss of mitochondrial membrane potential and increased intracellular free Ca^2+^ concentration (Xu et al., 2017). Wang et al. (2012) applied the “combination principle” in drug discovery and used several effective antitumor ingredients of Shi Quan Da Bu Wan as starting materials to synthesize TMP derivatives, which showed antiproliferative activities against HCT-8, Bel-7402, BGC-823, A-549, and A2780 human cancer cell lines and suppressed normal angiogenesis (Wang et al., 2012).

## 6 Clinical trials in tumor treatment

To overcome the clinical applications of TMP in tumor treatment, clinical trials remain essential. Most of the aforementioned treatment modalities of tumors involving TMP remain on the cellular and animal stage. Data obtained from human experiments remain limited (Table 1). Yang et al. (2010) administered intravenous TMP infusions to 56 patients with NHL in conjunction with chemotherapy with an application dose of 5 mg/kg and a maximum dose of 400 mg/day. Chen et al. (1997) combined 80 mg of TMP and 5% GS for intravenous infusion to affect platelet function and the coagulation state in 38 patients with lung cancer at a rate of 60–70 drops/min. These two studies suggest the potential use of TMP as an antitumor agent. Research on the antitumor effects of TMP requires the cooperation of various research organizations, which will promote the development of TMP-related drugs, improve its safety and treatment outcomes.

**TABLE 1:** Clinical application of tetramethylpyrazine (TMP) in cancer therapy.

S. no	Research target	Treatment	Research indicator	Effect
1	38 patients with lung cancer	80 mg of TMP added to 5% GS for intravenous infusion	Activation, adhesion, gathering and release of platelet; plasma VII C, vWF, Fg	Positive
2	56 patients with NHL	5 mg/kg a day intravenous TMP infusions	MDR and overexpression of P-glycoprotein (P-gp)	Positive

## 7 Conclusion and future perspectives

**FIGURE 3:**
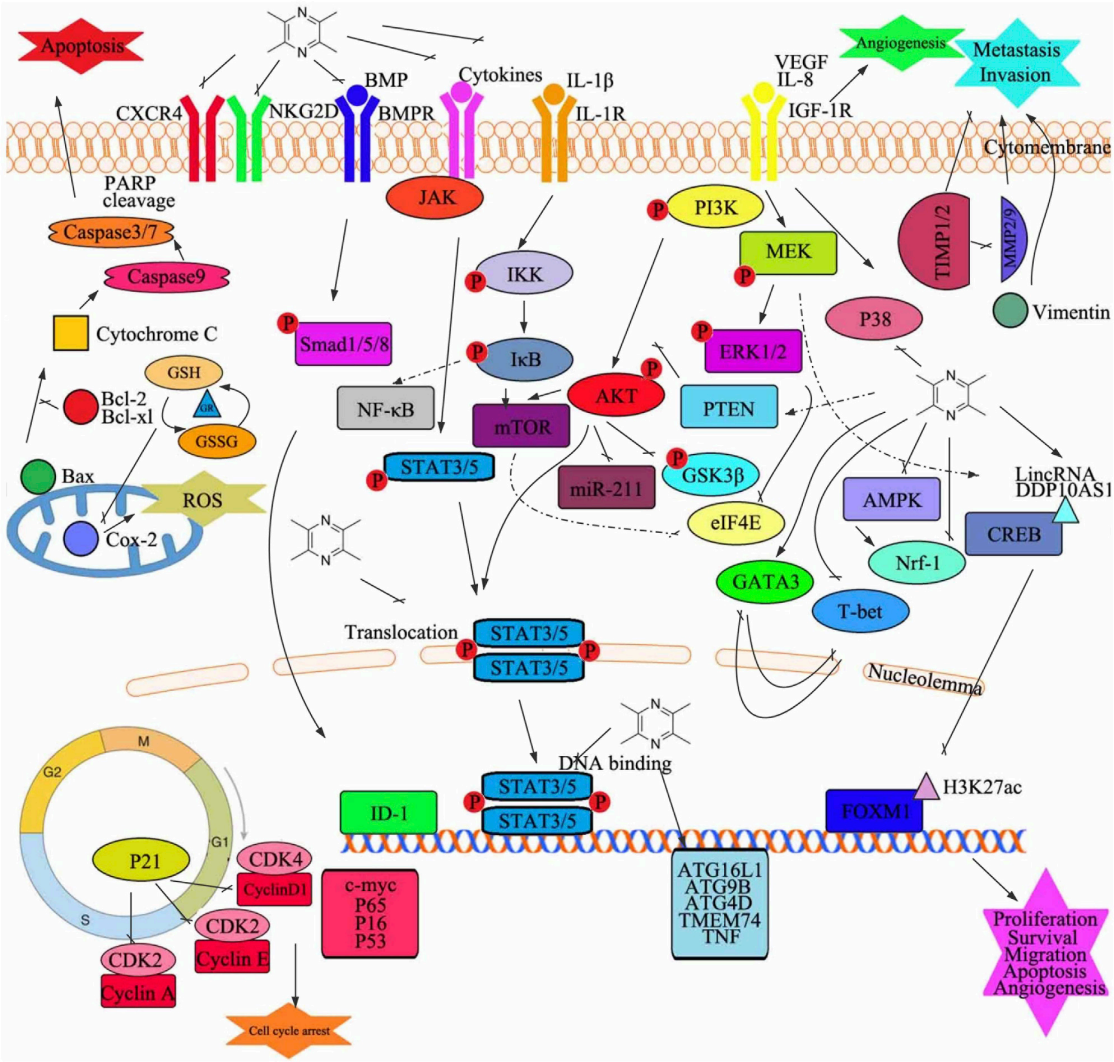
Graphical summary of the antitumor mechanisms underlying the role of tetramethylpyrazine. Tetramethylpyrazine acts on multiple signaling pathways in cancer cells to modulate several changes in phenotype such as cell proliferation, apoptosis, cell cycle arrest, migration, invasion, and angiogenesis. GSH, glutathione; GR, glutathione reductase; Bax, Bcl2-associated X protein; GSSG, oxidized glutathione; Cox-2, cytochrome c oxidase subunit II; ROS, reactive oxygen species; P, phosphorylation; NF-κB, nuclear factor kappa B; STAT, signal transducer and activator of transcription; CXCR4, C-X-C motif chemokine receptor 4; NKG2D, killer cell lectin-like receptor K1; BMP, bone morphogenetic protein; BMPR, bone morphogenetic protein receptor; JAK, Janus kinase; IL, interleukin; PARP, poly(ADP-ribose) polymerase; VEGF, vascular endothelial growth factor; IGF-1R, insulin-like growth factor 1 receptor; IKK, I-kappaB kinase; mTOR, mechanistic target of rapamycin kinase; miR, microRNA; PI3K, phosphatidylinositol 3-kinase; ERK, extracellular signal-regulated kinase; MEK, mitogen-activated protein kinase; PTEN, phosphatase and tensin; AKT, protein kinase B; GSK3β, glycogen synthase kinase 3 beta; eIF4E, eukaryotic translation initiation factor 4E; GATA3, GATA-binding protein 3; T-bet, T-box transcription factor 21; Nrf-1, nuclear respiratory factor 1; CREB, DNA-binding transcriptional regulator CreB; AMPK, protein kinase AMP-activated catalytic subunit alpha 1; MMP, matrix metalloproteinase; TIMP, tissue inhibitors of metalloproteinase; ID-1, inhibitor of DNA binding 1; FOXM1, forkhead box M1; ATG4D, autophagy-related 4D cysteine peptidase; TMEM74, transmembrane protein 74; TNF, tumor necrosis factor; c-myc, transcriptional regulator Myc-like; CDK, cyclin-dependent kinases; ATG16L1, autophagy-related 16 like 1; ATG9B, autophagy-related 9B

Several preclinical and clinical studies have independently verified that TMP exhibits chemical prevention and treatment potential for various cancers. Therefore, based on the above results, we can suggest factors that have led to the antitumor activity of TMP (Figure 3 and Table 2). However, there remains a need for sufficient evidence to continue to study the exact antitumor mechanism underlying the role of TMP and promote its clinical application in the treatment of cancer. Therefore, future research should focus on elucidating the precise anticancer mechanism underlying the role of TMP. Studies should continue to investigate MDR and TMP from various aspects and targets, to explain their mechanisms, as well as to evaluate the safety and effectiveness of TMP to facilitate its clinical application.

**TABLE 2:** Antitumor effect of tetramethylpyrazine against the tumor models of multiple organs.

S. no	Research target	Molecular target	Mode of action	Reference
Brain and nervous system				
1	Glioma cell line (C6) and gliomas transplanted into rat brains	Glutamate-induced increase in intracellular calcium	Proliferation, neuronal damage, and migration	Fu et al. (2008)
2	Glioma cell line (C6) and rats implanted with C6 cells	CXCR4	Migration, proliferation, colony formation, and S-phase arrest; tumor growth and microcirculation	Yu et al. (2012)
3	Glioma cell line (C6) and cerebral neurocytes	CXCR4	Inhibition and neural protection	Chen et al. (2013)
4	Umbilical vein endothelial cell line (ECV304), corneal neovascularization, and pulmonary fibrosis in rat model	SDF-1/CXCR4 axis	Neovascularization, fibrosis, and thrombosis	Cai et al. (2014)
5	Medulloblastoma cell line (Daoy)	MiR-211, PI3K/AKT, and mTOR pathways	Proliferation, migration, and invasion	Xu et al. (2018)
6	Rat pheochromocytoma-derived cell line (PC12)	Bcl-2, Bax, cytochrome c, and caspase-3	Apoptosis	Cheng et al. (2007)
Respiratory system				
7	Lung cancer cell line (A549, 95D)	Caspase-3 and Bax/Bcl-2	Proliferation, apoptosis, cell cycle arrest (S phase)	Huang et al. (2018)
8	Lung cancer cell line (A549) and metastatic nude mouse model	COX-2 and MMP-2/TIMP-2	Proliferation, cell cycle arrest, invasion, and metastasis	Zheng et al. (2012)
9	Lung cancer cell line (A549), microvascular endothelial cell line (HMEC-1), A549 xenograft in nude mice	BMP/Smad/Id-1 pathway	Proliferation, migration, angiogenesis, and tumor growth	Jia et al. (2016)
10	Lung cancer patients and PBMCs	Th2 type cytokines, T-bet/GATA3	PBMC	Wei et al., 2002 and 2004
11	Lung cancer cell line (PGCL3 and PAa)	No mention	Adhesion and invasion	Zhang et al. (1999)
12	Advanced cases of lung carcinoma	PAdT, PagT, VIII:C, dWF, and Fg	Metastasis	Chen et al. (1997)
Urinary system				
13	Prostate cancer cells (PCa cells)	DPP10-AS1/CBP/FOXM1 signaling pathway	Cell viability and apoptosis	Zhou et al. (2020)
14	Hormone-refractory prostate cancer cell line (PC-3)	EIF4E, mTOR, and MEK/ERK signaling pathways	Proliferation and apoptosis	Han et al. (2015)
15	Prostate cancer cell line (PC-3)	FOXM1	Proliferation, migration, and invasion	Zhou et al. (2017)
16	Renal cell carcinoma cell line (ccRCC)	NKG2D pathway, NKG2DLs, MICA/B, E-cadherin, vimentin, and fibronectin	Cell viability, proliferation, apoptosis, invasion, migration, and EMT	Luan et al. (2016)
17	Bladder carcinoma cells (T24)	Glutathione metabolism and glycerophospholipid metabolism	Angiogenesis, proliferation, and metastasis	Cui et al., 2019 and 2020
Blood and immune system				
18	T-cell leukemia cell line (SKW-3)	ICAM-1 and LFA-1	Adhesion	Zhao et al. (2000)
19	Leukemia cell line (HL-60)	C-myc, p27, CDK2, and cyclinE1	Differentiation, proliferation, and cell cycle arrest	Wu et al. (2011)
20	Acute lymphoblastic leukemia cell line (Jurkat and SUP-B15)	GSK-3β, NF-κB, and c-myc	Proliferation, apoptosis, and cell cycle arrest	Wang et al. (2015)
21	Leukemia cell line (U937)	Bcl-2, caspase-3	Proliferation, apoptosis, and cell cycle arrest	Wang et al. (2015)
22	Non-Hodgkin’s lymphoma (NHL) patients	P-glycoprotein (P-gp)	MDR	Yang and Jiang, (2010)
Digestive system				
23	Rats with DEN-induced HCC	Mitochondrial apoptotic pathway; Akt and ERK pathway	Tumor growth, apoptosis, and cell cycle arrest(G2/M)	Cao et al. (2015)
24	Mice with hepatic precancerous lesions	Serum marker enzymes, bile canaliculi hyperplasia	Pharmacokinetic variation and hepatoprotective effects	Feng et al. (2014)
25	HCC cell line (HepG2)	P53, Bcl-2/Bax protein ratio, cytochrome c, and caspase	Proliferation, mitochondrial apoptosis, cell cycle arrest (G0/G1 phase)	Bi et al. (2016)
26	HCC cell line (HepG2) and xenograft tumor models	Caspase-3 and PARP	Proliferation, autophagy, apoptosis, and ROS	Cao et al. (2015)
27	HCC cell line (HepG2)	IL-1R1/IκB/IKK/NF-κB signaling pathway	Invasion and migration	Wang et al. (2020)
28	HCC cell line (HepG2)	GSH/GSSG	Intracellular glutathione balance disruption and cytotoxicity	Ishida et al. (2012)
29	Gastric cancer cell line (SGC7901)	ROS, AMPK, cytochrome c, caspase-9, caspase-3, and mitochondrial membrane potential	Apoptosis	Yi et al. (2013)
30	Gastric cancer cell line (SGC-7901)	NF-xBp65, cyclinD1, and p16	Proliferation and apoptosis	Ji et al. (2014)
31	Colorectal cancer cell lines (SW480 and CT26)	P53-dependent mitochondrial pathway	Apoptosis, cell cycle arrest (G0/G1)	Bian et al. (2021)
32	Colorectal cancer cell line (HCT-116) and tumor-bearing mice	EMT(TGF-β1) and Wnt/β-catenin pathway (p-Akt, p-GSK-3β)	ROS, proliferation, migration, invasion, heteroadhesion, EMT, tumor growth, and lung metastasis *in vivo*	Zou et al. (2018)
Reproductive system				
33	Breast cancer cell line (MDA-MB-231) and xenograft tumors in nude mice	No mention	Proliferation, apoptosis, and cell cycle arrest (G0/G1)	Pan et al. (2015)
34	Breast cancer cell line (MDA-MB-231)	Akt and caspase-3	Proliferation, apoptosis, migration, and invasion	Shen et al. (2018)
35	Human breast cancer cell lines (MCF-7, MDA-MB-231), murine mammary carcinoma cell line (4T1), and 4T1 tumor-bearing mouse model	STAT3	Proliferation, migration, and tumor growth	Fan et al. (2021)
36	Triple-negative breast cancer cell line (MDA-MB-231)	Heparanase	Angiogenesis and autophagy	Li et al. (2021)
37	Ovarian cancer (OC) cell line (SK-OV-3 and OVCAR-3)	MiR-211	Proliferation, migration, and invasion	Zhang et al. (2021)
38	Ovarian carcinoma cell line (SKOV3)	IL-8 and ERK1/2, p38, and AP-1 pathways	Invasion and migration	Yin et al. (2011)
Other organs				
39	Melanoma cell line (B16F10) spontaneous metastasis model	CD34 and VEGF	Angiogenesis and metastasis	Chen et al. (2009)
40	UVA-induced melanoma/keratinocyte coculture system	TRP1, MITF, MAPK, TNFα, IL-1β, IL-8, and GM-CSF	Melanin content, cell viability, and cytokines release	Yeom et al. (2014)
41	Retinoblastoma cell line (WERI-Rb1), WERI-Rb1 cells injected into the eyes of athymic nude mice	Nrf-1 and CXCR4	Proliferation, cell cycle arrest(G1-phase), and tumor growth	Wu et al., 2017 and 2019
42	Osteosarcoma cell line (MG-63, SAOS-2, and U2OS), xenograft tumor mouse model	NF-κB, p65, BCL-2, and cyclin D1	Proliferation, apoptosis, cell cycle arrest (G0/G1), tumor growth	Wang et al. (2013)
43	Pharyngeal squamous cell line (FADU), HeLa, Hep G2, MCF-7, and A549	Depolarization of mitochondrial membrane potential	Proliferation, apoptosis, cell cycle arrest (S phase)	Wang et al. (2019)
Adriamycin				
44	HCC cell line (HepG2/ADM)	P-gp170 and MDR1	MDR	Mei et al. (2004)
45	HCC cell line (BEL-7402/ADM)	P-glycoprotein, MDR1, MRP2, MRP3, and MRP5	MDR	Wang et al. (2010)
46	Breast cancer cell line (MCF-7/ADR)	No mention	MDR	Zhou et al. (2007)
47	Breast cancer cell line (MCF-7/dox)	P-glycoprotein (P-gp)	MDR	Zhang et al. (2012)
48	Breast cancer cell line (MCF-7/A) and tumor xenografts *in vivo*	GSH, GSTπ, and JNK	Proliferation, ADR resistance, MDR	Zhang et al. (2014)
49	Breast cancer cell line (MCF-7/A)	EGFR/PI3K/Akt pathway	ADR resistance, apoptosis	Chen et al. (2014)
50	Human myelogenous leukemia cell line (K562/A02)	GSTπ	MDR	Song et al. (2011)
51	Breast cancer tissue samples and breast cancer cell lines (MCF-7 and T47D)	JAK2/STAT3 pathway	Epirubicin resistance	Liu et al. (2020)
Cisplatin				
52	Lewis lung cancer mice (nonsmall cell lung cancer)	VEGF, KLF4, and ADAMTS1	Tumor growth and angiogenesis	Tang et al. (2017)
53	Lung cancer cell line (A549, SPC-A-1, and LTEP-G-2) and implanted human lung cancer in mice	TrxR/Trx system, NF-κB, AKT, and ERK signaling pathways	MDR, ROS, apoptosis, tumor growth	Ai et al. (2016)
54	Bladder cancer cell line (Pumc-91/ADM and T24/DDP)	MRP1, GST, BCL-2, and TOPO-II	MDR and cell cycle arrest	Wang et al. (2016)
Paclitaxel				
55	Lung cancer cell line (A549)	VEGF, MMP2, TGF-β1, and E-cadherin	Metastasis	Xie et al. (2019)
56	Ovarian cancer cell line (A2780 and SKOV3) and A2780-heterografted BALB/c nude mice	ERK1/2 and Akt pathways	Angiogenesis, apoptosis, proliferation, and migration	Zou et al. (2019)
T-OA				
57	S180 mice (sarcoma)	NF-κB/p65 and COX-2	Pharmacokinetic evaluation and antitumor activity	Wang et al. (2013)
58	HCC cell line (Bel-7402)	NF-κB/p65 and COX-2	Apoptosis	Zhang et al. (2015)
59	HCC cell line (HepG2) and HT-29, HeLa, and BGC-823	No mention	Cytotoxicity, apoptosis, and nephrotoxicity	Chu et al. (2014)
DT-010				
60	Breast cell line (MCF-7)	GRP78	Dox-induced toxicity, apoptosis	Wang et al. (2016)
61	Breast cancer cell line (MCF-7/ADR)	P53, P-glycoprotein, and mitochondrial complex II	Proliferation, apoptosis, glycolysis, mitochondrial function, and metabolic process	Zhou et al. (2019)
62	Breast cancer cell line (MCF-7 and MDA-MB-231)	Mitochondrial complex II	Proliferation, cell cycle arrest, ROS generation, and mitochondrial dysfunction	Wang et al. (2016)
CSTMP				
63	Lung cancer cell line (A549)	IRE1α-TRAF2-ASK1 complex, ER stress, and JNK activation	Proliferation, cell cycle arrest, mitochondria-dependent apoptosis	Zhang et al. (2016)
64	Myeloma cell line (RPMI8226)	CHOP, GRP78, GRP94, cleaved caspase-12, PERK-eIF2α, IRE1α, and ATF6	Apoptosis, ER stress, and mitochondrial dysfunction	Sun et al. (2016)
